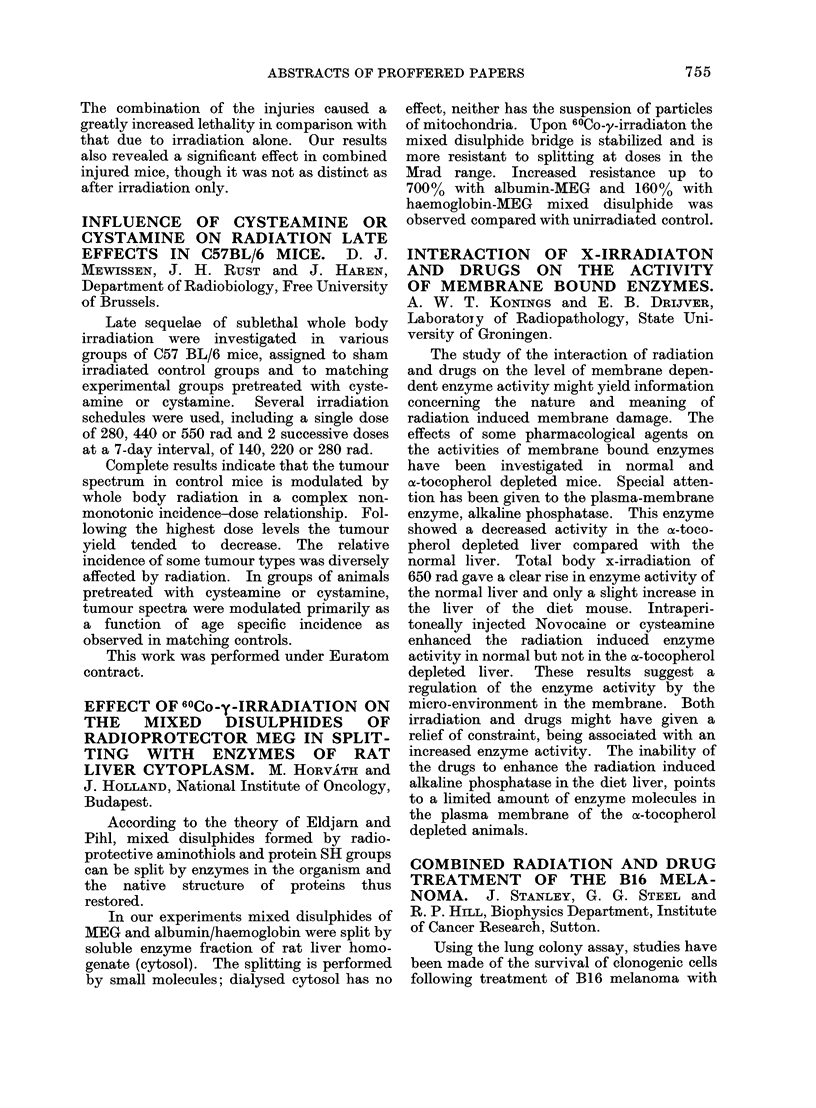# Proceedings: Effect of 60Co-gamma-irradiation on the mixed disulphides of radioprotector MEG in splitting with enzymes of rat liver cytoplasm.

**DOI:** 10.1038/bjc.1975.301

**Published:** 1975-12

**Authors:** M. Horváth, J. Holland


**EFFECT OF $^{60}$Co-$\gamma$-IRRADIATION ON THE MIXED DISULPHIDES OF RADIOPROTECTOR MEG IN SPLITTING WITH ENZYMES OF RAT LIVER CYTOPLASM.** M. HORVÁTH and J. HOLLAND, National Institute of Oncology, Budapest.

According to the theory of Eldjarn and Pihl, mixed disulphides formed by radioprotective aminothiols and protein SH groups can be split by enzymes in the organism and the native structure of proteins thus restored.

In our experiments mixed disulphides of MEG and albumin/haemoglobin were split by soluble enzyme fraction of rat liver homogenate (cytosol). The splitting is performed by small molecules; dialysed cytosol has no effect, neither has the suspension of particles of mitochondria. Upon $^{60}$Co-$\gamma$-irradiaton the mixed disulphide bridge is stabilized and is more resistant to splitting at doses in the Mrad range. Increased resistance up to 700% with albumin-MEG and 160% with haemoglobin-MEG mixed disulphide was observed compared with unirradiated control.